# Enhancing vaccine half-life as a novel strategy for improving immune response durability of subunit vaccines

**DOI:** 10.1371/journal.ppat.1012845

**Published:** 2025-01-08

**Authors:** Zhaoling Shen, Cheng Li, Wenping Song, Litong Liu, Yu Kong, Ailing Huang, Qingui Bao, Tianlei Ying, Yanling Wu

**Affiliations:** 1 Key Laboratory of Medical Molecular Virology (MOE/NHC/CAMS) and Shanghai Institute of Infectious Disease and Biosecurity, Shanghai Frontiers Science Center of Pathogenic Microorganisms and Infection, School of Basic Medical Sciences, Fudan University, Shanghai, China; 2 College of Life Sciences, Hebei Agricultural University, Baoding, China; 3 Fosun Diagnostics (Shanghai) Co., Ltd, Shanghai, China; 4 Shanghai Engineering Research Center for Synthetic Immunology, Shanghai, China; The University of Texas Medical Branch at Galveston, UNITED STATES OF AMERICA

## Abstract

Vaccines are widely regarded as one of the most effective strategies for combating infectious diseases. However, significant challenges remain, such as insufficient antibody levels, limited protection against rapidly evolving variants, and poor immune durability, particularly in subunit vaccines, likely due to their short in vivo exposure. Recent advances in extending the half-life of protein therapeutics have shown promise in improving drug efficacy, yet whether increasing in vivo persistence can enhance the efficacy of subunit vaccines remains underexplored. In this study, we developed two trimeric SARS-CoV-2 subunit vaccines with distinct pharmacokinetic profiles to evaluate the impact of vaccine persistence on immune efficacy. A self-assembling trimeric subunit vaccine (RBD-HR/trimer) was designed, followed by an extended-persistence variant (RBD-sFc-HR/trimer) incorporating a soluble monomeric IgG1 fragment crystallizable. We demonstrated that RBD-sFc-HR/trimer elicited more robust and higher levels of neutralizing antibodies, with potent and broad neutralization activity against multiple SARS-CoV-2 variants. Notably, RBD-sFc-HR/trimer induced a durable immune response, significantly increasing the number of memory B cells and T cells. This study provides critical insights for designing vaccines that achieve potent and long-lasting immune responses against infectious diseases.

## Author summary

Vaccines are critical for combating infectious diseases and offer protection against a wide range of pathogens. However, significant challenges remain, such as insufficient antibody levels, limited protection against rapidly evolving variants, and poor immune durability, particularly in subunit vaccines, likely due to their short *in vivo* exposure. To develop long-acting vaccines, we design two SARS-CoV-2 vaccines (RBD-sFc-HR/trimer and RBD-HR/trimer) with distinct pharmacokinetic profiles to evaluate the impact of vaccine persistence on immune efficacy. The results shown that RBD-sFc-HR/trimer elicited more robust and higher levels of neutralizing antibodies, with potent and broad neutralization activity against multiple SARS-CoV-2 variants. Notably, RBD-sFc-HR/trimer induced a durable immune response, significantly increasing the number of memory B cells and T cells. This study provides critical insights for designing vaccines that achieve potent and long-lasting immune responses against infectious diseases.

## Introduction

Vaccines are critical for combating infectious diseases and offer protection against a wide range of pathogens [1]. Although multiple vaccines have been developed, several challenges remain, including insufficient antibody levels, limited breadth of protection against rapidly evolving variants and poor durability of immunity. Generally, critical proteins displayed on the viral surface, which are involved in virus entry, serve as potent candidates for subunit vaccine design because they can induce specific neutralizing antibodies that effectively prevent viral invasion [2–4]. However, these protein-based vaccines often suffer from a short half-life after administration due to rapid systemic clearance. In contrast, natural infections expose antigens to the immune system over extended periods, eliciting potent humoral immune responses [5]. Recently, the sustained delivery of vaccines via surgically implantable osmotic pumps to maintain serum concentrations has been demonstrated to increase vaccine efficacy [6]. Nonetheless, concerns remain regarding the safety, stability and time tunability of such vaccines, which may hinder further development. Therefore, there has been significant interest in developing long-acting vaccines.

Currently, prolonging the half-life of protein-based therapeutics has been shown to significantly increase drug efficacy [7–9], owing to their increased *in vivo* circulation time and concentration. In our previous studies, the benefits of half-life extension were demonstrated in traditional protein-based therapeutics and adeno-associated virus (AAV)-delivered gene therapy [5,10]. However, the impacts of vaccine persistence *in vivo*, particularly those of subunit vaccines, on the immune response and protection against infectious diseases have received little attention.

In this study, as a proof-of-concept to evaluate whether enhancing vaccine persistence can improve immune efficacy, we designed two trimeric SARS-CoV-2 subunit vaccines with distinct in vivo pharmacokinetic profiles. First, a self-assembling trimeric subunit vaccine (RBD-HR/trimer) was generated by fusing the receptor-binding domain (RBD) and heptad-repeat sequences 1 and 2 (HR1/HR2) from the SARS-CoV-2 spike protein [11–13]. The HR1/HR2 regions automatically assembled into a 6-helix bundle structure. Next, to enhance the *in vivo* persistence, we engineered another vaccine by fusing RBD-HR/trimer with a soluble monomeric fragment crystallizable (single-chain Fc, sFc) from IgG1, which retained a human FcRn binding profile and half-life *in vivo* comparable to that of dimeric Fc [14,15]. Moreover, we demonstrated that RBD-sFc-HR/trimer possesses high druggability with rapid purification and homogeneity, conferring complete RBD exposure and strong binding to ACE2 and neutralizing antibodies. Importantly, RBD-sFc-HR/trimer exhibited an increased half-life in mice, increasing to 33.05 h compared with 6.22 h for RBD-HR/trimer. While both vaccines induced RBD-specific IgGs, RBD-sFc-HR/trimer elicited higher levels of neutralizing antibodies than did RBD-HR/trimer, demonstrating more potent binding and neutralization activity against various SARS-CoV-2 variants, including Alpha, Beta, Gamma, Delta, Lambda, and Omicron. By day 162 after vaccination, RBD-specific neutralizing antibody titers in the RBD-HR/trimer group had declined, whereas those in the RBD-sFc-HR/trimer group remained stable, indicating that durable humoral immune responses were induced by RBD-sFc-HR/trimer. Thus, extending the *in vivo* persistence of vaccines to ensure prolonged exposure to the immune system has emerged as a highly promising approach to achieving sustained and robust levels of neutralizing antibodies. This study provides insights and guidance for vaccine design to achieve potent and long-lasting immune responses against infectious diseases.

## Results

### Design and characterization of the recombinant RBD-sFc-HR/trimer protein

**Fig 1:**
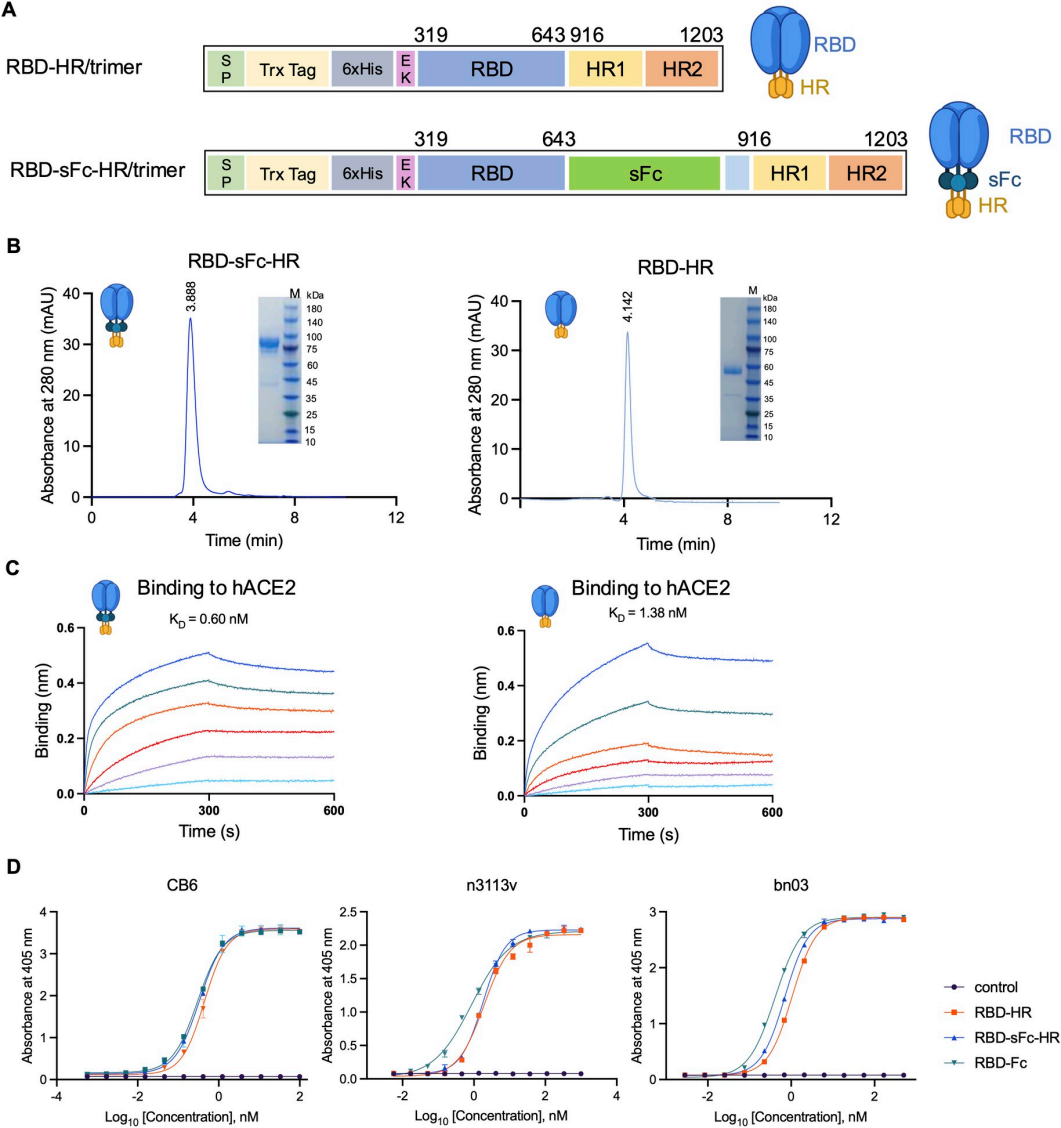
Design and characterization of the recombinant RBD-sFc-HR/trimer protein. **(A)** Schematic view of the RBD-sFc-HR/trimer protein and the control protein RBD-HR/trimer. The RBD-sFc-HR/trimer includes an RBD (319–643 aa) sequence derived from wild-type SARS-CoV-2, the soluble monomeric immunoglobulin G 1 (IgG1) fragment crystallizable (sFc) and two heptapeptide repeats (HR1, amino acids L916–P1162; HR2, amino acids D1163–L1203) of the SARS-CoV-2 S2 subunit. **(B)** HPLC analysis of the recombinant RBD-sFc-HR/trimer and RBD-HR/trimer. The insets show the SDS-PAGE analyses of the eluted protein samples. mAU, milli-absorbance units; M, marker. **(C)** Biolayer interferometry (BLI) binding assay between the purified receptor ACE2 and RBD-sFc-trimer (left) or RBD-HR/trimer (right). **(D)** Enzyme-linked immunosorbent assays (ELISAs) for binding assays between our purified proteins (RBD-HR/trimer, RBD-sFc-HR/trimer, RBD-Fc, MSL109 (control)) and RBD-specific antibodies (CB6, n3113v, bn03). The data represent the means ± SDs.

In a previous study, the RBD region of the spike protein and self-assembling HR1/HR2 were linked to develop a trimeric protein vaccine against SARS-CoV-2 [13]. To generate long-acting RBD trimers, we utilized an engineered sFc from IgG1, previously developed through directed evolution and high-throughput screening by our group [14,15]. The RBD was fused directly to the N-terminus of the sFc, and at its C-terminus, it was linked to the two heptapeptide repeats (HR1 and HR2) of the S2 subunit via a C-terminal (G_4_S)_3_ linker (Fig 1A), resulting in the construct designated RBD-sFc-HR/trimer. Owing to the nature of the HR1-HR2 six-helix bundle, the RBD-sFc-HR protein self-assembles to form a native-like trimeric structure. SDS-PAGE analysis revealed that the RBD-sFc-HR/trimer and RBD-HR/trimer were reduced to monomers of approximately 85 kDa and 55 kDa, respectively. The intact RBD-sFc-HR/trimer, with a molecular mass of 255 kDa, eluted at ~3.89 min on HPLC, while RBD-HR/trimer eluted at ~4.14 min, confirming their trimeric structures (Fig 1B). Importantly, RBD-sFc-HR/trimer is a simple purification process that uses protein G affinity chromatography without the need for a purification tag, resulting in a highly purified product with a single peak observed via HPLC.

Next, we tested the binding of the RBD-sFc-HR/trimer protein to human ACE2, the cellular receptor for the RBD, via ForteBio biolayer interferometry (BLI). The results showed that RBD-sFc-HR/trimer and RBD-HR/trimer had similarly high affinities for ACE2 (Fig 1C), which is consistent with the findings of previous studies [13,16,17]. We also investigated the binding of the RBD-sFc-HR/trimer protein to RBD-specific binding antibodies, including n3113v, bn03, and CB6, which have been reported in previous studies [18,19]. The RBD-sFc-HR/trimer displayed strong binding to these antibodies, with EC_50_ values of approximately 1.81 nM, 0.70 nM, and 0.33 nM, respectively, comparable to those of RBD-HR/trimer (Fig 1D). Our results suggested that our antigen design maintained proper RBD folding and exposed the receptor-binding motif.

### Extended *in vivo* half-life of RBD-sFc-HR/trimer via pH-dependent FcRn binding

**Fig 2:**
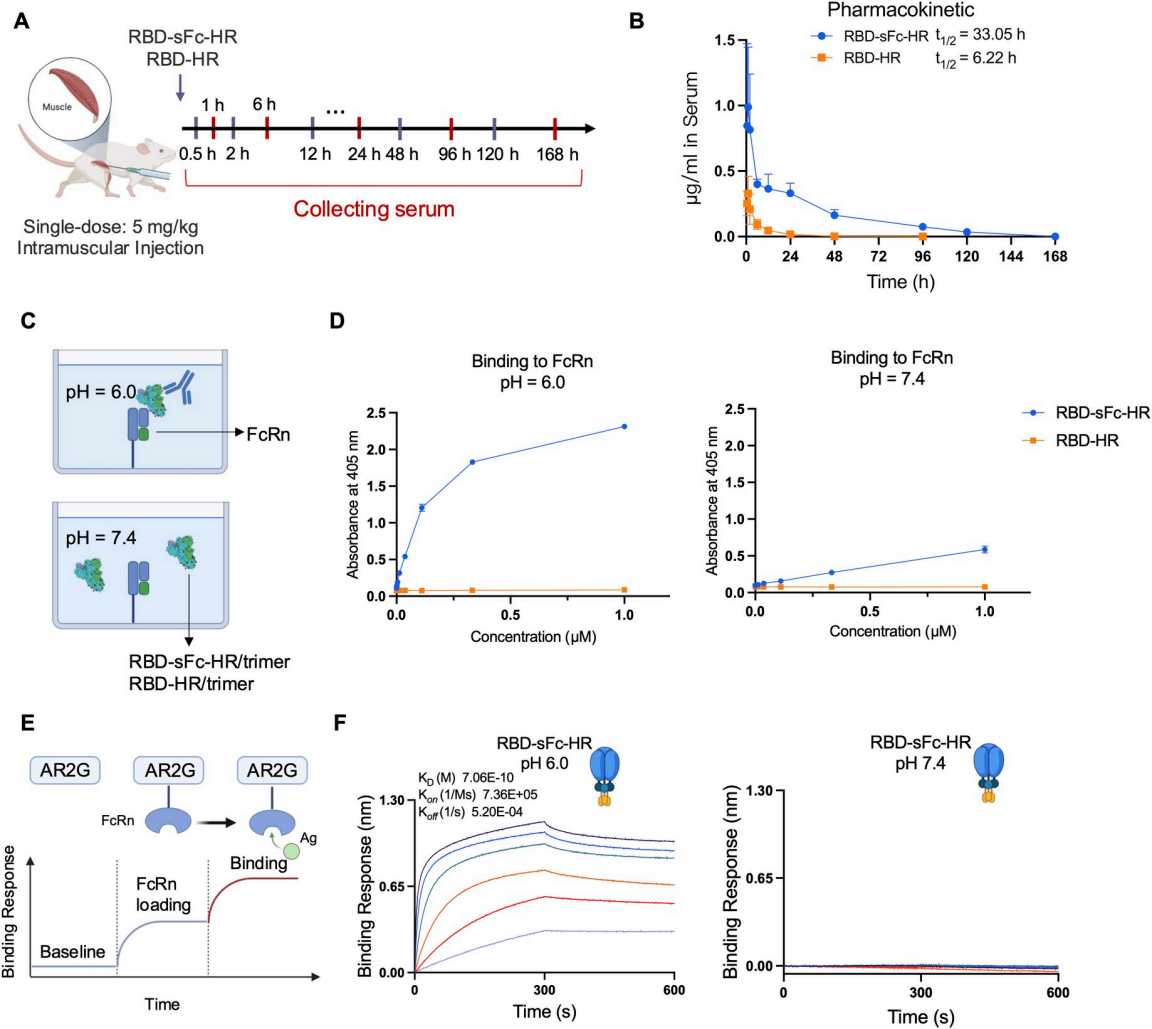
Extended half-life of the recombinant RBD-sFc-HR/trimer protein. **(A)** For the RBD-sFc-HR/trimer or RBD-HR/trimer proteins, 5 mg/kg was intramuscularly injected, and the time points at which the serum was collected were recorded. Created with BioRender.com. **(B)** Pharmacokinetics of RBD-sFc-HR/trimer (blue) and RBD-HR/trimer (orange) in mice (n = 5), as measured by ELISA. The data represent the means ± SDs. **(C)** Schematic view of the RBD-sFc-HR/trimer protein binding to FcRn by ELISA. **(D)** ELISA for binding assays between RBD-HR/trimer and RBD-sFc-HR/trimer and FcRn at pH 6.0 and pH 7.4. The data represent the means ± SDs. **(E)** Schematic view of the RBD-sFc-HR/trimer protein binding to FcRn by BLI. **(F)** BLI binding assay between purified receptor FcRn and RBD-sFc-trimer or RBD-HR/trimer at pH 6.0 and pH 7.4.

To examine whether sFc fusion can prolong the *in vivo* circulating half-life of the trimer, we determined the pharmacokinetics of RBD-sFc-HR/trimer and RBD-HR/trimer in mice. The RBD-sFc-HR/trimer and RBD-HR/trimer were intramuscularly injected into the mice at a single dose of 5 mg/kg, and blood was collected for concentration detection (Fig 2A). The results clearly revealed that RBD-sFc-HR/trimer exhibited a significantly improved serum half-life of more than 33 h, which was 5.31-fold longer than that of RBD-HR/trimer in mice (Fig 2B). According to the pharmacokinetic profile analysis, the RBD-sFc-HR/trimer group presented a significantly lower clearance rate (CL), higher maximal concentration (Cmax), and greater total exposure over time (AUCinf) than did the RBD-HR/trimer group (S1 Fig).

The extended plasma half-life of therapeutic proteins by IgG1 Fc-fusion is primarily mediated by pH-dependent binding to FcRn. At acidic pH (~pH 6.0) in the endosome, Fc binds to FcRn, protecting the proteins from degradation. The complex then returns to the cell surface, where it is released into circulation at physiological pH (~pH 7.4) [15,20,21]. This pH-dependent binding profile, tightly at acidic pH but weakly at physiological pH, is crucial for prolonging the half-life of the Fc-fusion protein [22]. Therefore, we tested the binding of RBD-sFc-HR/trimer and RBD-HR/trimer to FcRn at pH 6.0 or 7.4 via ELISA and BLI (Fig 2C and 2E). The results demonstrated that the RBD-sFc-HR/trimer tightly bound to FcRn at acidic pH (pH 6.0) with high affinity (Fig 2D and 2F), but no binding activity was observed at physiological pH (Fig 2D and 2F), which was identical to that of sFc alone (S2 Fig). In contrast, the RBD-HR/trimer displayed no FcRn binding at either acidic or physiological pH values (Fig 2D).

### Potent humoral immune response and broad neutralization activity against SARS-CoV-2 variants elicited by RBD-sFc-HR/trimer

**Fig 3:**
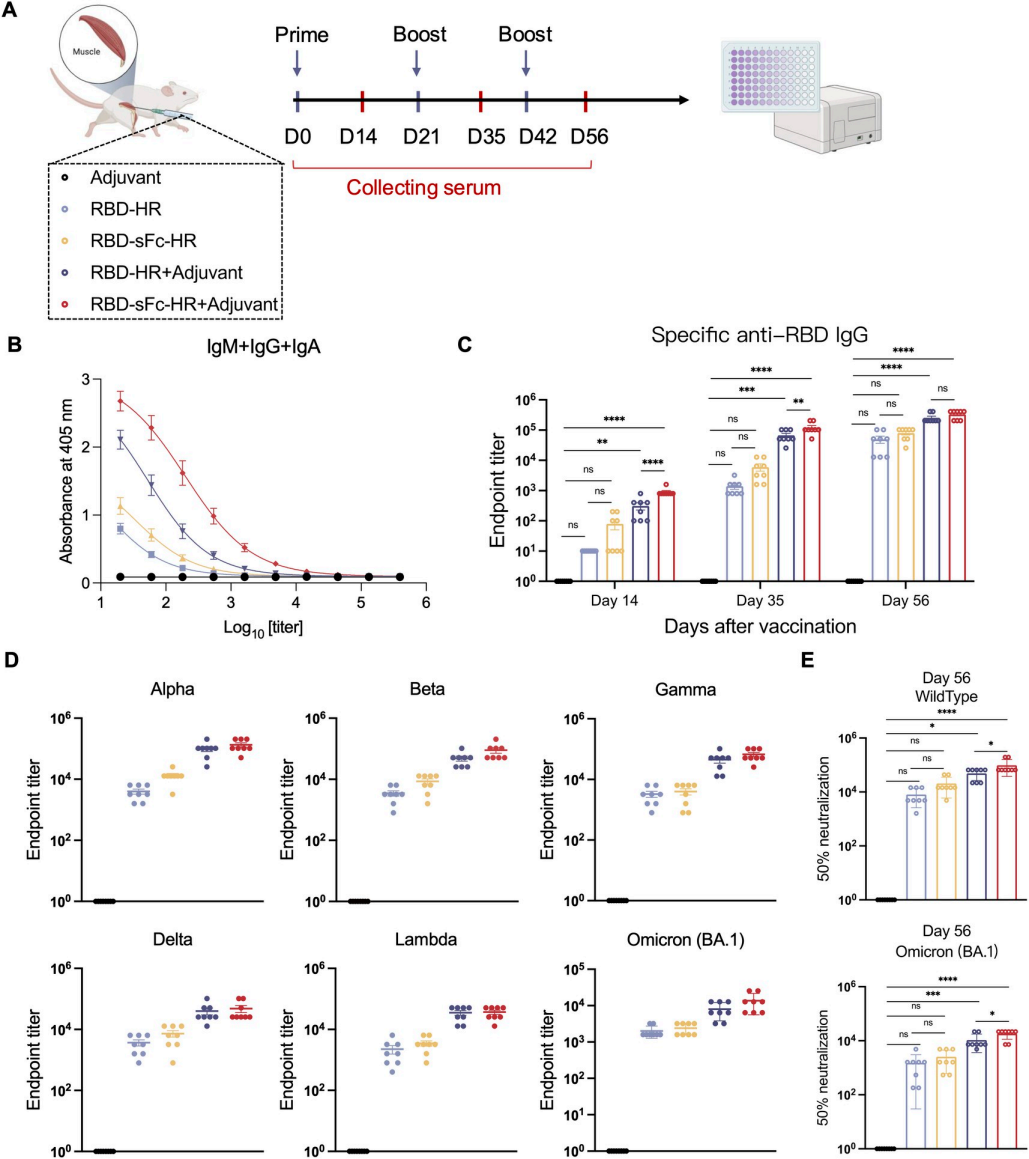
The RBD-sFc-HR/trimer vaccine elicited a potent humoral immune response and broad neutralization activity against SARS-CoV-2 variants. **(A)** Schematic representation of the mouse immunization protocol. Serum samples were obtained from the mice at 14 d after each immunization. Created with BioRender.com. **(B)** Serum was collected on d 14 after the prime vaccination. The levels of IgM+IgG+IgA against the RBD protein were detected via a series of serum dilutions. The data represent the means ± SDs. **(C)** An assay of the endpoint titers of specific anti-RBD IgGs induced by our vaccine was performed via ELISA (n = 8 mice per group). **(D)** Serum was analyzed for IgG antibodies against recombinant RBDs from multiple variants of SARS-CoV-2 (Alpha, Beta, Delta, Lambda, Gamma, and Omicron) via ELISA (n = 8 mice per group). The absorbance at 405 nm was measured via a microplate reader with the wavelength correction set to 630 nm. **(E)** Neutralization of SARS-CoV-2 pseudovirus (wild-type or Omicron) infection in Huh-7 cells by immune serum (D 56). The data are presented as the means ± SDs. *P* values were determined via one-way ANOVA. A *P*-value < 0.05 was considered statistically significant (*P < 0.05; **P < 0.01; ***P < 0.001; ****P < 0.0001).

The immunogenicity of the RBD-sFc-HR/trimer and RBD-HR/trimer vaccines was evaluated in mice (n = 8 per group) via intramuscular injection of 10 μg of protein formulated with or without the MF59-like adjuvant (AddaVax) on day 0, 21, and 42. Blood samples were collected at 14 days after each immunization (Fig 3A). The adjuvant-only group served as the negative control. We first determined the RBD-specific antibody titers via ELISA. The total anti-RBD IgM+IgG+IgA antibody and RBD-specific IgG antibody levels in the serum of the RBD-sFc-HR/trimer group were greater than those in the RBD-HR/trimer group, regardless of adjuvant addition (Figs 3B and S3A). In contrast, the levels of RBD-specific IgM antibody induced by both RBD-HR/trimer or RBD-sFc-HR/trimer were moderate on day 14, and no RBD-specific IgA was detected in any of the groups (S3A Fig). Importantly, the RBD-sFc-HR/trimer vaccine induced a faster RBD-specific IgG antibody response than the RBD-HR/trimer vaccine did. By day 35, after two immunizations, the specific anti-RBD IgG endpoint titer of the RBD-sFc-HR/trimer group had reached 10^5, whereas the RBD-HR/trimer group required a third immunization to achieve comparable levels. The specific anti-RBD IgG endpoint titer of the RBD-sFc-HR/trimer with AddaVax on day 56 was 3.33 × 10^5, which was 1.30-fold higher than that of the RBD-HR/trimer, indicating that the RBD-sFc-HR/trimer vaccine provides a stronger and more rapid immune boost (Fig 3C). In addition, we tested the specific anti-S2 IgG endpoint titer of the RBD-sFc-HR/trimer with AddaVax on day 56, which was 2.48 × 10^3, 1.65-fold higher than that of the RBD-HR/trimer (S3B Fig).

Antibodies specific to the SARS-CoV-2 RBD are associated with potent viral neutralization. Serum samples were analyzed for the binding of induced IgG antibodies to multiple variants of SARS-CoV-2, including Alpha, Beta, Delta, Lambda, Gamma, and Omicron. Our findings indicated that RBD-sFc-HR/trimer immunization induced antibodies with strong binding activity against these SARS-CoV-2 variants, and this activity was greater than that in the RBD-HR/trimer group (Fig 3D). The specific anti-RBD IgG endpoint titer of the RBD-sFc-HR/trimer with AddaVax to Alpha, Beta, Delta, Lambda, Gamma, and Omicron were 134,400, 89,600, 48,000, 36,800, 67,200 and 13,600, respectively. Pseudovirus neutralization assays were conducted with sera from immunized mice. On day 56, the RBD-sFc-HR/trimer vaccine elicited high titers of neutralizing antibodies against wild-type SARS-CoV-2, which were significantly greater than those induced by the RBD-HR/trimer vaccine (Fig 3E). However, against the Omicron variant, the neutralizing potency of the RBD-sFc-HR/trimer vaccine was reduced but remained greater than that of the RBD-HR/trimer vaccine, possibly due to the numerous mutations in the Omicron variant and the use of the wild-type RBD in our vaccine design (S4 Fig).

### Durable antibody responses induced by the RBD-sFc-HR/trimer vaccine

**Fig 4:**
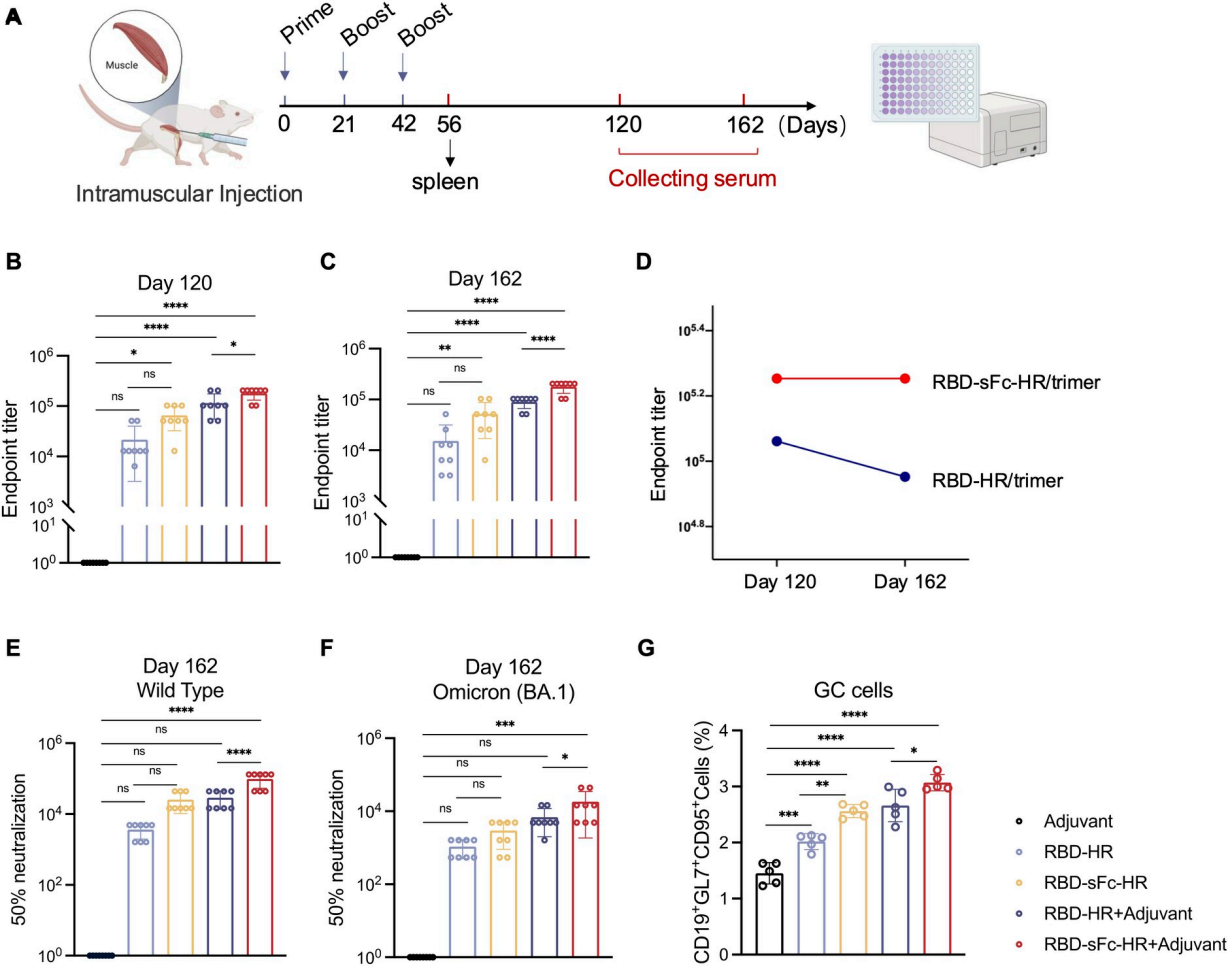
The RBD-sFc-HR/trimer vaccine induced durable neutralizing antibody responses and promoted GC B-cell generation. **(A)** Schematic representation of the mouse immunization protocol. Serum samples were obtained from the mice at 14 d after each immunization. Created with BioRender.com. **(B)** Levels of specific anti-RBD IgGs at the endpoint titer on d 120. **(C)** Levels of specific anti-RBD IgG antibodies at the endpoint titer on d 162. The absorbance at 405 nm was measured via a microplate reader with the wavelength correction set to 630 nm. **(D)** Temporal profile of specific anti-RBD IgG levels from d 120 to d 162. **(E-F)** Neutralization of SARS-CoV-2 pseudovirus (wild-type or Omicron) infection in Huh-7 cells by immune serum (d 162). n = 8 mice in each group in B-E. **(G)** The frequency of germinal center B cells (CD3^-^CD19^+^GL7^+^CD95^+^) in the spleen (n = 5). The data are presented as the means ± SDs. *P* values were determined via one-way ANOVA. A *p* value < 0.05 was considered statistically significant (*P < 0.05; **P < 0.01; ***P < 0.001; ****P < 0.0001).

To assess the long-term immune responses of the RBD-sFc-HR/trimer and RBD-HR/trimer vaccines, we monitored 8 mice per group for 120 days and 162 days postimmunization (Fig 4A). On day 120, the RBD-sFc-HR/trimer group presented significantly higher endpoint titers than did the RBD-HR/trimer group (Fig 4B). This trend continued on day 162, with the RBD-sFc-HR/trimer group maintaining higher endpoint titers, both with and without adjuvant (Fig 4C). Specifically, the endpoint titers of anti-RBD IgG between the RBD-sFc-HR/trimer with AddaVax and the RBD-HR/trimer with AddaVax showed an increase by 1.56-fold and 2.00-fold, on day 120 and 162, respectively. The endpoint titers in the RBD-sFc-HR/trimer group remained stable from day 120 to 162, whereas the titers in the RBD-HR/trimer group declined over the same period (Fig 4D). Similar results were observed in the neutralization assays. The RBD-sFc-HR/trimer vaccine also maintained relatively high titers of neutralizing antibodies in mice against SARS-CoV-2 wild-type and Omicron pseudoviruses, showed a 3.36- and 2.65-fold increase, respectively, compared to RBD-HR/trimer vaccine (Fig 4E and 4F). These findings indicate that the humoral immune responses induced by the RBD-sFc-HR/trimer vaccine were largely preserved over 4 months.

In addition, germinal center (GC) B cells were evaluated by measuring the percentage of CD19^+^GL7^+^CD95^+^ cells in the spleen (S4 Fig). Compared with the RBD-HR/trimer vaccine, the RBD-sFc-HR/trimer vaccine induced a significantly greater GC B-cell response, with a 1.15-fold increase (Fig 4G). Importantly, in the nonadjuvant groups, the difference was more significant, approximately 1.26-fold.

### The RBD-sFc-HR/trimer vaccine elicited a robust T-cell response

**Fig 5:**
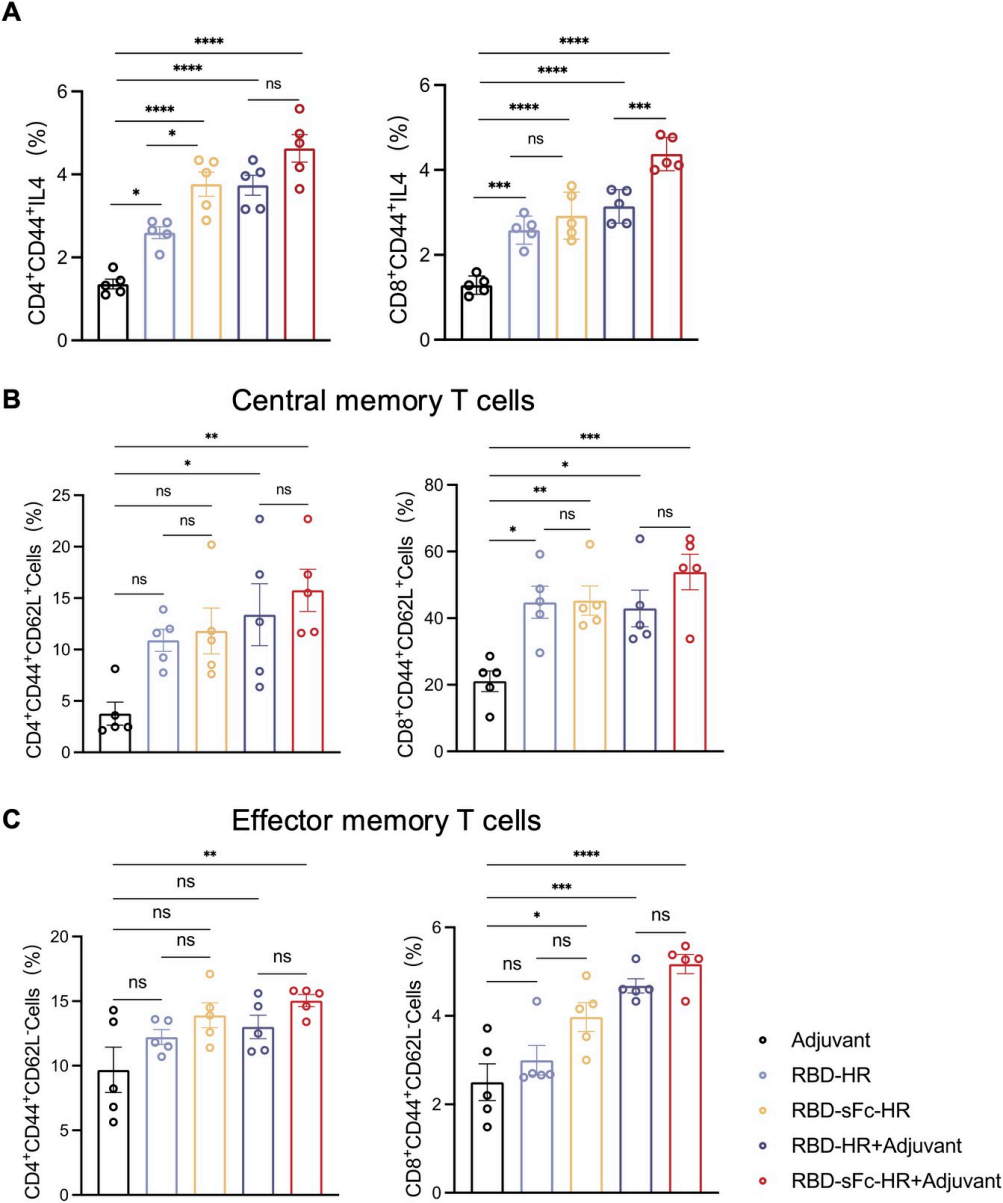
The RBD-sFc-HR/trimer vaccine elicited a robust T-cell response *in vivo*. **(A)** The number of RBD-specific IL-4-producing memory T cells in the spleen was analyzed by flow cytometry after stimulation with the SARS-CoV-2 spike RBD peptide pool for three d. Memory T cells were gated on CD3^+^CD4^+^CD44^+^ (left) or CD3^+^CD8^+^CD44^+^ (right) populations. **(B)** The percentage of central memory (CD44^+^CD62L^+^) CD4^+^ or CD8^+^ T cells in the spleen. **(C)** The percentage of effector memory (CD44^+^CD62L^-^) CD4^+^ or CD8^+^ T cells in the spleen. n = 5 mice in each group in A-C. Spleen samples were obtained from the mice at 14 d after the third immunization. The data are presented as the means ± SEMs. *P* values were determined via one-way ANOVA. A *p* value < 0.05 was considered statistically significant (*P < 0.05; **P < 0.01; ***P < 0.001; ****P < 0.0001).

Cellular immune responses play a key role in the outcome of infectious diseases. Therefore, we further determined the ability of the designed vaccines to activate T-cell responses. The immunized mice were sacrificed for lymphocyte isolation from the spleen. The robust T-cell response induced by the RBD-sFc-HR/trimer vaccine significantly increased the percentages of RBD-specific IL-4-producing memory T cells, including CD3^+^CD4^+^CD44^+^IL4^+^ and CD3^+^CD8^+^CD44^+^IL4^+^ cells, compared with those in the RBD-HR/trimer vaccine and adjuvant-only groups (Figs 5A and S5). Memory T cells are divided into effector memory and central memory populations according to their function and phenotype [23]. Specifically, the RBD-sFc-HR/trimer with adjuvant group presented the highest percentages of effector memory (CD3^+^CD4^+^CD44^+^CD62L^-^ or CD3^+^CD8^+^CD44^+^CD62L^-^) and central memory (CD3^+^CD4^+^CD44^+^CD62L^+^ or CD3^+^CD8^+^CD44^+^CD62L^+^) T cells, indicating a robust effector memory response (Fig 5B and 5C).

## Discussion

Compared with other types of vaccines, such as inactivated or live-attenuated vaccines, recombinant protein vaccines offer significant advantages, including high safety due to the absence of viral genome integration and cost-effectiveness, making them strong candidates for widespread use. This study highlights the design and development of a protein-based vaccine with enhanced *in vivo* persistence, demonstrating its ability to elicit robust and enduring neutralizing antibody responses. Our innovative approach involves fusing a soluble monomeric Fc region from IgG1 to the protein vaccine, aiming to prolong the plasma half-life and improve the exposure of immunodominant neutralizing epitopes, thereby better-stimulating B cells and enhancing cellular immune responses.

Increasing the half-life of protein-based drugs, particularly through the Fc-fusion strategy, notably improved their *in vivo* activity and therapeutic efficacy. In recent years, the Fc fusion approach has become an important backbone for vaccine design because of its rapid purification, extended half-life, and increased immunogenicity of target antigens [24]. Fc-fused protein vaccines, such as those developed for MERS, SARS-CoV, H5N1 influenza, and SARS-CoV-2, have shown greater immunogenicity than their non-Fc-fused counterparts [25–29]. This enhanced immunogenicity is largely attributed to the dimer format enabled by Fc-fusion [26]. In this study, we selected a self-assembling trimeric subunit vaccine for SARS-CoV-2. The sFc, developed by mutation of critical residues located on the IgG1 Fc dimerization interface, is a monomeric form with half the molecular weight of dimeric Fc but maintains a similar *in vivo* half-life [15]. Notably, the sFc exhibits no binding activity against Fc receptors, including FcγRI (CD64), FcγRIIa (CD32a), and FcγRIIIa (CD16a). Therefore, sFc fusion allows RBD-HR/trimer to maintain the trimeric structure and an improved half-life *in vivo* through pH-sensitive binding of sFc to FcRn [22]. Importantly, this fusion facilitates rapid purification, thereby streamlining the production process and ensuring consistency in vaccine quality.

*In vivo* studies demonstrated that the RBD-sFc-HR/trimer vaccine provided a stronger immune boost for generating both RBD- and HR-targeting antibodies compared to the RBD-HR/trimer and adjuvant groups. Previous studies have shown that the HR domains in the S2 subunit are highly conserved among coronaviruses and elicit potent cross-neutralizing antibodies against SARS-CoV-2 including Omicron sublineages [30]. Furthermore, the native-like trimeric structure developed by this immunogenicity enhancement strategy improves the presentation of conserved epitopes on RBD, which are targeted by cross-neutralizing antibodies [31]. As a result, immunization with the RBD-HR/trimer and RBD-sFc-HR/trimer vaccines elicited broadly neutralizing antibodies against SARS-CoV-2 and its variants, including Alpha, Beta, Gamma, Delta, Lambda and Omicron. The breadth of neutralization induced by this vaccine should be further monitored in future studies.

Importantly, to investigate whether the neutralizing antibody response in mice immunized with RBD-sFc-HR/trimer could maintain a high level for a longer period, we tested the binding ability and neutralization activity of serum from mice immunized with 10 μg RBD-sFc-HR/trimer with adjuvant at 162 days post-1^st^ immunization. The results showed that serum elicited high titers of neutralizing antibodies in mice capable of neutralizing both wild-type and Omicron pseudoviruses, indicating the potential of the vaccine for long-term protection, which may contribute to cost-effectiveness by reducing the need for frequent immunizations.

In summary, the RBD-sFc-HR/trimer vaccine exhibits a prolonged half-life and robust immune responses, making it a potential candidate for effective and long-lasting protection against SARS-CoV-2 and its variants. Integrating half-life extension into vaccine design offers a novel approach to improving vaccine efficacy and durability, paving the way for future advancements in vaccine development for infectious diseases.

## Materials and methods

### Ethics statements

Female BALB/c mice aged 6–8 weeks were purchased from the Shanghai Model Organisms Center and maintained under specific pathogen-free (SPF) conditions. All procedures related to animal handling, care, and treatment were performed and approved by the Ethics Committee of the School of Basic Medical Sciences at Fudan University in accordance with the recommendations in the Guide for the Care and Use of Laboratory Animals of Fudan University.

### Cell culture

293T cells were obtained from the American Type Culture Collection (ATCC). Huh-7 cells were obtained from the Cell Bank of the Chinese Academy of Sciences (Shanghai, China). The cells were cultured in Dulbecco’s modified Eagle’s medium (DMEM) supplemented with 10% fetal bovine serum (FBS) at 37°C in a 5% CO_2_ atmosphere.

### RBD-sFc-HR/trimer protein design and plasmid construction

The RBD-sFc-HR/trimer recombinant protein is constructed from the RBD of the wild-type isolate of SARS-CoV-2 (C538S), the single-chain Fc (sFc) and two heptapeptide repeats (HR1, amino acids L916—L966; HR2, amino acids K1157—L1203) of the SARS-CoV-2 S2 subunit. Specifically, the RBD and sFc are directly fused in-frame, while the sFc is connected to the HR region via a C-terminal (G_4_S)_3_ linker. The RBD-HR/trimer is constructed with a direct linkage between the RBD and HR. This designed protein sequence is further fused at its N-terminus in a tandem manner with the sequences of a heavy chain signal peptide of the antibody to guarantee protein secretion, a Trx tag to help protein folding, a 6xHis tag to facilitate protein purification, and an enterokinase (EK) cleavage site for tag removal. The gene sequence described above was first amplified via PCR and then incorporated into the pcDNA3.1 vector via the BmtI and HindIII restriction sites.

### Protein expression and purification

The RBD-sFc-HR/trimer proteins were expressed via the Expi293T Expression System and purified with Protein G resin (GenScript). The resin was collected on a chromatography column, washed with PBS (pH 7.4), and eluted in 0.1 M glycine (pH 2.7). The eluates were equilibrated with 1 M Tris-HCl (pH 9.0), and the buffer was immediately replaced with PBS (pH 7.4). RBD-HR was purified by Ni-NTA (Yeasen). The proteins were sterilely filtered and stored at −80°C until use. Protein integrity was analyzed by sodium dodecyl sulfate–polyacrylamide gel electrophoresis (SDS-PAGE). The wild-type RBD (WT) was produced and stored in our laboratory. His-tagged Omicron RBDs were purchased from AcroBiosystems, and other RBD variants (including alpha, beta, gamma, lambda, and delta) were purchased from Sino Biological, Inc.

### Size-exclusion high-performance liquid chromatography (SEC-HPLC)

Thirty micrograms of RBD-sFc-HR/trimer or RBD-HR/trimer were applied to a TSK-Gel Super G200 (TSK-GEL) using a Waters AQUITY UPLC H-class system. The mobile phase was PBS buffer (pH 7.4) run at a flow rate of 0.4 ml/min. The absorbance was monitored at 280 nm. The UV traces were analyzed and integrated by the area under the curve (AUC) to determine the percentages of aggregation, monomers and degradants.

### SDS-PAGE analysis

For denaturing SDS-PAGE, 10 μl of protein sample (30 μg protein) was mixed with 2.5 μl of 5× sample loading buffer (Yeasen) and heated at 100°C for 10 min. These samples were then loaded into precast 4%-12% gradient gels (Yeasen) with 8 μl of prestained SDS-PAGE standards. Electrophoresis was performed at room temperature for approximately 1 h at a constant voltage (140 V) in running buffer until the dye front reached the end of the gel. The gels were stained with Coomassie Brilliant Blue (Yeasen) and imaged.

### Binding affinity determination via a biolayer interferometry (BLI) binding assay

The kinetics of the binding of the RBD-sFc-HR/trimer and RBD-HR/trimer to human ACE2 were determined on the OctetRED96 device (Sartorius). Human ACE2 (Novoprotein, #C419) at 10 μg/ml buffered in sodium acetate (pH 5.0) was immobilized onto activated AR2G biosensors (Sartorius) and then incubated with threefold serial dilutions of 500 nM RBD-sFc-HR/trimer or RBD-HR/trimer in kinetics buffer for 300 s after a short baseline step. The sensors were transferred into a kinetics buffer to allow a 300 s dissociation step.

The kinetics of the binding of the RBD-sFc-HR/trimer to FcRn were determined on the OctetRED96 device (Sartorius). FcRn at 15 μg/ml buffered in sodium acetate (pH 5.0) was immobilized onto activated AR2G biosensors (Sartorius) and then incubated with threefold serial dilutions of 500 nM RBD-sFc-HR/trimer in kinetics buffer (pH 6.0 or pH 7.4 PBS containing 0.02% Tween-20) for 300 s after a short baseline step. The sensors were transferred into a kinetics buffer (pH 6.0 or pH 7.4 PBS containing 0.02% Tween-20) to allow a 300 s dissociation step.

Data analysis software was used for curve fitting with a 1:3 or 1:2 binding model to calculate equilibrium dissociation constant values. K_D_ values were determined with R^2^ values greater than the 98% confidence level.

### Enzyme-linked immunosorbent assays (ELISAs) for binding

RBD-HR and RBD-sFc-HR at 100 ng per well were coated in a 96-well half-area microplate (Corning #3690) at 4°C overnight for the ELISA binding assay. The wells were washed three times with PBS containing 0.05% Tween-20 (PBST) and blocked with 100 μl of PBS containing 3% skim milk for 1 h at 37°C. The wells were subsequently washed three times with 0.05% PBST. Fifty microliters of threefold serially diluted protein in 1% milk-PBS were added, and the mixture was incubated at 37°C for 90 min. The plate was subsequently washed with 0.05% PBST three times. Anti-Flag-HRP (Sigma-Aldrich) was used for the detection of bn03 and n3113v, and anti-Fab-HRP (Sigma–Aldrich) was used for the detection of the IgG antibody (CB6). After incubation at 37°C for 45 min, the plates were washed five times with 0.05% PBST, and the absorbance at 405 nm was measured after incubation with ABTS substrate (Invitrogen) for 15 min. The data were plotted via GraphPad Prism, and the antibody concentration or plasma dilution was transformed into log[concentration]/[dilution] for four-parameter nonlinear regression fitting. The EC_50_ (concentration for 50% of the maximal effect) and ED_50_ (median effective dose) were calculated.

FcRn at 100 ng per well was coated in a 96-well half-area microplate (Corning #3690) at 4°C overnight for the ELISA binding assay. The wells were washed three times with PBS containing 0.05% Tween-20 (PBST) and blocked with 100 μl of PBS containing 3% skim milk for 1 h at 25°C. The wells were subsequently washed three times with 0.05% PBST. Fifty microliters of threefold serially diluted protein in 0.05% PBST (pH 6.0 or pH 7.4) was added, and the mixture was incubated at 37°C for 90 min. The plate was subsequently washed with 0.05% PBST (pH 6.0 or pH 7.4) three times. Anti-Flag-HRP was used for the detection of RBD-HR/trimer, and anti-Fc-HRP was used for RBD-sFc-HR/trimer. After incubation at 37°C for 45 min, the plates were washed five times with 0.05% PBST (pH 6.0 or pH 7.4), and the absorbance at 405 nm was measured after incubation with ABTS substrate (Invitrogen) for 15 min. The data were plotted via GraphPad Prism.

### Pharmacokinetic assay in mice by intramuscular injection

For the RBD-sFc-HR/trimer or RBD-HR/trimer proteins, 5 mg/kg was injected intramuscularly. Mice (n = 5 per group) and serum were collected via ocular veniplex at 30 min, 1, 2, 6, and 12 h, and every 24 h for each mouse, followed by centrifugation and serum collection. The supernatant was harvested and stored at -80°C for further quantification.

The plasma protein concentrations were determined via ELISA. ACE2 at 100 ng per well was coated in a 96-well half-area microplate. Anti-Flag-HRP was used to detect RBD-HR/trimer, and anti-Fc-HRP was used to detect RBD-sFc-HR/trimer for the ELISA binding assay. The data were plotted via GraphPad Prism.

### Vaccination and serum collection

MF59-like adjuvant (AddaVax) was used according to a previous report [13]. The RBD_WT_-sFc-HR/trimer protein was mixed with the MF59-like adjuvant at a volume ratio of 1:1. The mice (n = 8 per group) were immunized via intramuscular injection on d 0, 21, and 42. Each group of mice was immunized with 50 μl of adjuvant, 10 μg of RBD_WT_-HR/trimer protein, 10 μg of RBD_WT_-sFc-HR/trimer protein, 10 μg of RBD_WT_-HR/trimer protein with an equal volume of adjuvant, or 10 μg of RBD_WT_-sFc-HR/trimer protein with an equal volume of adjuvant. Adjuvants were applied as a sham control.

A preimmunization blood sample was collected via an ocular veniplex before the prime vaccination and was collected on day 14 after each vaccination. After standing for 1 h, the serum samples were obtained via centrifugation at 16°C and 3,500 rpm/min for 20 min before being stored at -80°C until use.

### Enzyme-linked immunosorbent assays (ELISAs) for measurement of RBD-specific antibody responses

RBD-Fc or SARS-CoV-2 S2 protein (Acro) at 100 ng per well was coated in a 96-well half-area microplate (Corning #3690) overnight at 4°C. The wells were washed three times with PBS containing 0.05% Tween-20 (PBST). The antigen-coated plate was blocked with PBS containing 3% skim milk for 1 h at 37°C and washed three times with PBST (PBS with 0.05% Tween 20). Fifty microliters of threefold serially diluted proteins in 1% milk-PBS or plasma from vaccinees at a dilution was added, and the mixture was incubated at 37°C for 90 min. The plate was subsequently washed with PBST three times. The anti-mouse-IgG-Fab-HRP (Abcam #ab98659), anti-mouse-IgM-HRP (ABclonal), anti-mouse-IgA-HRP (Proteintech) and anti-mouse-IgM+IgA+IgG-HRP (Abcam #ab102448) antibodies used to measure the titers of specific IgG, IgM, IgA, and IgM+IgG+IgA, respectively, were diluted 1:5000 with 1% milk-PBS and added to the wells (50 μl/well). After incubation at 37°C for 45 min, the plates were washed five times with PBST, and the absorbance at 405 nm was measured after incubation with ABTS substrate (Invitrogen) for 15 min. The data were plotted via GraphPad Prism, and the antibody concentration or plasma dilution was transformed into log[concentration]/[dilution] for four-parameter nonlinear regression fitting. The endpoint titer was defined as the highest reciprocal dilution of serum that resulted in an absorbance greater than 2.5-fold greater than the background value [32].

### Construction of SARS-CoV-2 variant spike plasmids

The gene encoding the Omicron (strain BA.1) S protein was purchased from GenScript and then subcloned and inserted into the pcDNA3.1 vector. To generate plasmids encoding the SARS-CoV-2 spike proteins of various variants (alpha, beta, gamma, and delta), site-directed mutagenesis was performed via a QuickMutationÔ Site-Directed Mutagenesis Kit (Beyotime Biotechnology). The vector pcDNA3.1 encoding the S protein of SARS-CoV-2 from the Wuhan-Hu-1 strain (GenBank: MN_908947) was generated and served as a template. First, two complementary primers containing the desired mutation were designed. Following site-directed mutagenesis PCR, the mutated plasmid was amplified with staggered nicks, and DpnI restriction endonuclease (NEB) was added to digest the parental vector for approximately 3 h at 37°C. Afterwards, the PCR products, including the nicked vector with the desired mutations, were directly transformed into DH5α competent cells. The mutation was verified by DNA sequencing.

### Establishment and validation of a panel of SARS-CoV-2 pseudoviruses

To compare the neutralizing activity of vaccine sera against SARS-CoV-2, we produced HIV pseudotyped with different spike proteins as previously described [18]. Plasmids encoding the spike protein of WT and various variants in the pcDNA3.1 vector and the luciferase reporter-expressing HIV-1 backbone in pNL4-3.luc. RE were cotransfected 1:1 into 293T cells. After 8 h, the culture medium was replaced with fresh DMEM, and the cells were cultured for an additional 48 h. The supernatants containing the pseudoviruses were harvested via centrifugation at 3500 rpm/min for 10 min and filtered through a 0.45 μm pore size filter before being stored at 80°C in aliquots for use. Tenfold serially diluted pseudoviruses were used to infect Huh-7 cells for 12 h. The supernatant was refreshed 12 h post-infection, and the cells were cultured for an additional 48 h. Luciferase activity was recorded to determine the infectivity of the pseudovirus.

### Pseudovirus neutralization assay

Different pseudoviruses were used for neutralization assays to detect neutralizing antibodies in the sera of immunized mice. Huh-7 cells (10^4^/well) were coated in 96-well cell culture plates overnight to form monolayers of adherent cells. In accordance with the pseudoviral infectivity determination assay mentioned above, a viral dilution of the relative light unit (RLU) of approximately 30,000 was used. Briefly, serum samples from each group were subjected to three serial dilutions ranging from 20 to 131,220. 50 μl of diluted serum samples were co-incubated with equal volume of diluted pseudovirus for 1 h at 37°C. The mixture was added and incubated with Huh-7 cells for 10 h, after which the culture medium was replaced with fresh DMEM supplemented with 10% FBS. The cells were incubated for another 48 h to express luciferase. Finally, the cell supernatant was removed, the cells were washed with PBS twice, 100 μl of Bright-Lite Luciferase Assay Substrate from a luciferase kit (Vazyme) was added, and the RLU was detected with a multimode microplate reader (PerkinElmer, USA) with Kaleido 3.0 software.

The percentage of neutralization was calculated by the RLU measured in the pseudovirus control well (pseudovirus and cells) minus the RLU measured in the sample well (sample, virus, and cells) and dividing the RLU of the pseudovirus control well minus the cell control (cells alone) and multiplying by 100%. The 50% neutralizing titers of the pseudoviruses were expressed as the highest dilution that caused 50% neutralization relative to the average of the virus control wells and was calculated via a nonlinear regression model (inhibitor versus normalized response) in GraphPad Prism.

### Flow cytometry

Splenocytes were isolated 14 days after the third immunization and plated at 2 × 10^6^ cells per well in 24-well plates in complete medium (RPMI 1640 (Meilinbio, # MA0215) supplemented with 10% fetal bovine serum (FBS), containing IL-2 (Novoprotein, #CK24, 100 U/ml) and the SARS-CoV-2 spike RBD peptide pool (SinoBiological, #PP002, 5 μg/ml) to activate the cells at 37°C in a 5% CO_2_ atmosphere for 48 h in the presence of brefeldin A (5 μg/ml) for the last 4 h. Complete medium was used as a negative control. The cells were then stained with anti-CD3e AF700 (1:200, BD Pharmingen, #550583), anti-CD4 FITC (1:200, BD Pharmingen), anti-CD8 PE (1:200, BD Pharmingen), anti-CD44 BV421 (1:200, BD Pharmingen, #563970), anti-CD62L PE-Cy7 (1:200, BD Pharmingen, #560516), anti-CD19 BV510 (1:200, BD Pharmingen, #562956), anti-CD95 PE-Cy7 (1:200, BD Pharmingen, #557653), anti-GL7 Percp-Cy5.5 (1:200, Biolegend, #144610), fixed with Cytofix/Cytoperm (200 μl, BD Pharmingen, #554714), washed in 1 × Perm/Wash buffer (BD Pharmingen, #554714), and then stained with anti-IL-4 BV605 (1:200, BD Pharmingen, #564007) in Perm/Wash buffer for 40 min at 4°C, washed twice in Perm/Wash buffer and suspended in PBS with 2% FBS. Samples were acquired on a BD LSRFortessa multicolor flow cytometer and analyzed via FlowJo software.

### Quantification and statistical analysis

The results of antibody binding to the RBD via ELISA, RBD-sFc-HR affinity via BLI, and virus neutralization are presented as the mean values from three independent experiments. Data and statistical analyses were performed via GraphPad Prism 9.0. Flow cytometry data were analyzed via FlowJo 9.0. The *P* values were determined via one-way ANOVA, as indicated in each figure legend. Statistical significance was defined as a *P*-value < 0.05.

## Supporting information

S1 Fig The pharmacokinetic profile analysis of RBD-sFc-HR and RBD-HR, includes peak concentration (Cmax), time to peak (Tmax), Area Under Curve (AUCinf), plasma clearance (CL), half-life (t1/2). Created with BioRender.com.
(TIFF)

S2 Fig The BLI binding assay between purified receptor FcRn and sFc in pH 6.0 and pH 7.4. (TIFF)

S3 Fig (A) Serum was collected on day 14 after the prime vaccination. The levels of IgM, IgG and IgA against the RBD protein were detected via a series of serum dilutions. The data represent the means ± SDs. (B) The endpoint titers of specific anti-S2 IgGs at day 56 induced by vaccines were assessed via ELISA (n = 8 mice per group).
(TIFF)

S4 Fig Sequence alignment of SARS-CoV-2 RBD from multiple variants (Alpha, Beta, Delta, Lambda, Gamma, Omicron). Red region shows the conserved sequence.
(TIFF)

S5 Fig Germinal center B cells (CD3^-^CD19^+^GL7^+^CD95^+^), central memory (CD44^+^CD62L^+^) of CD4 or CD8 T cells, and effector memory (CD44^+^CD62L^-^) of CD4 or CD8 T cells in spleen. (A), (B) and (C) represented the typical FCM figures.
(TIFF)

S1 File All original data in the article, Figs 1–5 and S1–S5 Figs. (XLSX)
